# Mechanisms regulating the CRISPR-Cas systems

**DOI:** 10.3389/fmicb.2023.1060337

**Published:** 2023-02-28

**Authors:** Marta Zakrzewska, Michal Burmistrz

**Affiliations:** ^1^Department of Environmental Microbiology and Biotechnology, Faculty of Biology, Institute of Microbiology, University of Warsaw, Warsaw, Poland; ^2^Department of Molecular Microbiology, Biological and Chemical Research Centre, Faculty of Biology, University of Warsaw, Warsaw, Poland; ^3^Centre of New Technologies, University of Warsaw, Warsaw, Poland

**Keywords:** CRISPR-Cas, SgRNA, NAPs, Acr proteins, cAMP receptor protein, quorum Sensing, HtpG

## Abstract

The CRISPR-Cas (Clustered Regularly Interspaced Short Palindromic Repeats- CRISPR associated proteins) is a prokaryotic system that enables sequence specific recognition and cleavage of nucleic acids. This is possible due to cooperation between CRISPR array which contains short fragments of DNA called spacers that are complimentary to the targeted nucleic acid and Cas proteins, which take part in processes of: acquisition of new spacers, processing them into their functional form as well as recognition and cleavage of targeted nucleic acids. The primary role of CRISPR-Cas systems is to provide their host with an adaptive and hereditary immunity against exogenous nucleic acids. This system is present in many variants in both Bacteria and Archea. Due to its modular structure, and programmability CRISPR-Cas system become attractive tool for modern molecular biology. Since their discovery and implementation, the CRISPR-Cas systems revolutionized areas of gene editing and regulation of gene expression. Although our knowledge on how CRISPR-Cas systems work has increased rapidly in recent years, there is still little information on how these systems are controlled and how they interact with other cellular mechanisms. Such regulation can be the result of both auto-regulatory mechanisms as well as exogenous proteins of phage origin. Better understanding of these interaction networks would be beneficial for optimization of current and development of new CRISPR-Cas-based tools. In this review we summarize current knowledge on the various molecular mechanisms that affect activity of CRISPR-Cas systems.

## 1. Introduction

Prokaryotes inhabit various ecological niches and are often exposed to unfavorable environmental conditions. To survive, they have developed multiple cell protection mechanisms that are triggered by various stress factors which include, among others, exogenous nucleic acids that enter the bacterial cell by transduction, transformation, or conjugation. The foreign genetic material is detected by various mechanisms such as restriction and modification systems, nucleoid-associated proteins like H-NS, intracellular exonucleases as well as the CRISPR-Cas (Clustered Regularly Interspaced Short Palindromic Repeats - CRISPR associated proteins) systems. What makes the latter one unique is that it is an adaptive and hereditary immune system. In addition to providing immunity against foreign nucleic acids, it is involved in numerous biological processes, such as the regulation of gene expression or biofilm formation (Cui L. et al., 2020). The CRISPR-Cas systems have been identified in most of the Archaea domain species and about half species of the Bacteria domain (Grissa et al., 2007; Makarova et al., 2015). Because of this wide distribution there are many variants of these systems, classification of which is based on the sequence and organization of the *cas* genes and the sequence of repeats. There are two main classes named 1 and 2. In class 1 CRISPR-Cas systems, the effector complex consists of multiple subunits. On the contrary, class 2 systems, utilize the effector complexes consisting of a single multidomain protein. Both classes are further divided in types and subtypes. Class 1 has 3 types and 16 subtypes, whereas class 2 has 3 types and 17 subtypes (Makarova et al., 2015, 2020; Shmakov et al., 2015). Despite of the class and type of a given CRISRP-Cas system all of them utilize two main components: the genomic CRISPR array and the *cas* gene operon usually located in a close vicinity to the array. A single CRISPR locus consists of a noncoding leader sequence containing promoters and regulatory protein binding sites; followed by a series of direct repeat and spacer units. Identical direct repeats play a regulatory role, whereas unique spacers determine the specificity of the system (Ishino et al., 1987; Jansen et al., 2002). This specificity of CRISPR-Cas is acquired by spacers having sequence complimentary to fragments of mobile genetic elements. As spacer sequences are acquired during encounter with the mobile genetic elements, the CRISPR array becomes, in a way, an archive of previous infections. Cas proteins are essential for the functioning of the system during each of three distinct phases: adaptation, crRNA maturation and interference.

During the adaptation phase new spacer fragments are acquired from the exogenous nucleic acid. For many CRISPR-Cas types this is possible due to recognition of the protospacer adjacent (PAM) motif located in the direct vicinity of protospacers. Protospacer sequences are incorporated into a CRISPR matrix becoming spacers, which allow sequence recognition based on complementarity. PAM motifs themselves are not incorporated into the CRISPR locus to avoid subsequent autoimmunity and cleavage of the host genome. In types that recognize exogenous RNA, the role of PAMs is played by Protospacer Flanking Site (PFS). Type III systems do not utilize neither PAM, nor PFS. Instead, targeting own CRISPR array is prevented by interaction between crRNA and the repeat of the CRISPR matrix (Samai et al., 2015). The adaptation phase requires the participation of the Cas1 and Cas2 proteins (Nuñez et al., 2014). These two proteins often require additional Cas proteins for spacer acquisition (Nuñez et al., 2014; Vorontsova et al., 2015; Wei et al., 2015), i.e., Cas3, Cas4 and the effector complex for type I systems (Mohanraju et al., 2016). Involvement of Cas9, Csn2 and tracrRNA (transactivating crRNA, small RNA) has also been shown for the type II (Heler et al., 2015). In the case of some systems, like subtype III-B, the acquisition of RNA-derived spacers involves reverse transcriptase linked to the Cas1 protein (Silas et al., 2016).

The second phase, the maturation of the crRNA, begins with the transcription of CRISPR array initiated at the leader region, which leads to the production of a long RNA molecule called pre-crRNA, which is subsequently processed into separate crRNAs. The pre-crRNA of class 1 systems is typically processed by a dedicated Cas6 ribonuclease that trims it to produce a functional mature crRNA molecules, each of which contain single spacer sequence flanked by fragments of repeat sequences (Hochstrasser and Doudna, 2015). An exception from that being the I-C subtype, where trimming is performed by Cas5 (Mohanraju et al., 2016). In class 2 systems pre-crRNA processing is conducted by the effector proteins (Cas9, Cas12 and Cas13, for types II, V and VI, respectively). To obtain functional crRNA type II systems incorporate several factors such as proteins other than Cas, RNAse III and an unknown nuclease that performs transcript cleavage, as well as tracrRNA (Shmakov et al., 2015). TracrRNA is encoded in the vicinity of the *cas* genes and the CRISPR array and it is transcribed in parallel with the pre-crRNA transcript. It is characterized by the presence of sequences complimentary to the repeat-derived part of crRNA, which allows the formation of crRNA-tracrRNA duplexes, which are responsible for guidance of an effector protein (Chylinski et al., 2013; Zetsche et al., 2015). For some of the CRISPR-Cas systems it was shown that precise trimming of the pre-crRNA is not required for efficient targeting (Yan et al., 2019).

In the interference phase, mature crRNA becomes incorporated into the effector complex and participates in the search for sequences complimentary to the one coded by spacer fragment of crRNA. The class 1 crRNA-bound multiprotein complex, which is called CRISPR-associated complex for antiviral defense (Cascade), first recognizes the PAM sequence. Subsequent to PAM motif recognition, hybridization of the spacer-derived crRNA fragment with the protospacer results in the formation of an R loop in which the crRNA is paired with one of DNA strands (Jiang et al., 2016). The formation of the R-loop triggers the conformational changes of the complex to induce a Cas3 endonuclease which initiates cleavage of the target nucleic acid fragment, substrate for acquisition by the Cas1–Cas2 complex (Mohanraju et al., 2016). In the opposite to other class 1 CRISPR-Cas systems type III systems lack Cas3, they do not include Cas6 protein as an integral part of the effector complex, and they do not utilize PAM recognition. Instead, binding of crRNA effector complex to the target DNA causes cleavage through Cas10 cyclase, known as the large subunit of the effector complex. Cas10 generates cyclic oligoadenylates that activate RNase III and lead to non-specific degradation of RNA (Mohanraju et al., 2016). Type III systems can also specifically recognize single stranded RNAs through proteins of the Cas7 family. The RNA and DNA degradation reactions in these systems are coupled, ensuring targeting of actively transcribed phage DNA (Kazlauskiene et al., 2016). Class 2 CRISPR-Cas systems usually encompass all necessary activities within a single, multidomain protein which is involved in all the functional steps of CRISPR–Cas immunity. In the interference phase, the Type II Class 2 system uses the Cas9 protein complex, mature crRNA, and tracrRNA, which is unique for type II, to scan DNA for PAM motif. The recognition of the complementary sequence induces the formation of an R loop structure which ultimately generates a double strand cleavage by the HNH (His-Asn-His) and RuvC domains, with the first HNH domain responsible for cleaving the strand associated with the crRNA and RuvC cutting the second strand. A class 2 type VI-A effector protein, Cas13a, which interferes with single stranded RNA, has two HEPN domains (higher eukaryotes and prokaryotes nucleoide-binding domain) with RNase activity. Cas13a is responsible for both pre-crRNA maturation and global RNA degradation (East-Seletsky et al., 2016). The extensive description of mechanisms of CRISPR-Cas systems biology and mechanisms can be found here (Mohanraju et al., 2016; Burmistrz et al., 2020).

Due to the great complexity of CRISPR-Cas systems and the fact that their operation has or may have serious consequences, it can be assumed that the host will be able to control and, if necessary, fine tune the activity of these systems. Regulation of the CRISPR-Cas systems are a broad, still not fully understood topic. The regulatory mechanisms enable cell to “allocate” the available energy in a manner that is most beneficial the prokaryotic cell. Understanding the intricate network of connections between elements involved in regulation can contribute to an overall understanding of how exactly the CRISPR-Cas systems work. In addition to that, multiple biotechnological applications of the CRISPR-Cas systems can benefit from it as well. If regulatory mechanisms allow bacteria to save energy, they can also be used to save energy in biotechnological processes, making them more efficient and profitable. Furthermore, there are well known shortcomings of the CRISPR-Cas systems used in modern genetic engineering like off-target cleavage, cytotoxicity, and immune responses to name just a few. These could be reduced or even eliminated making CRISPR-Cas systems more precise and safer. Modulation of activity can occur during each stage of the CRISPR-Cas activity, both through components encoded in the CRISPR array and translation phase, as well as during the post-translation phase. The aim of this review is to present current state of knowledge about various mechanism involved in modulation of the CRISPR-Cas systems activity.

## 2. Regulation of CRISPR-Cas activity

### 2.1. Quorum sensing

**Figure 1:**
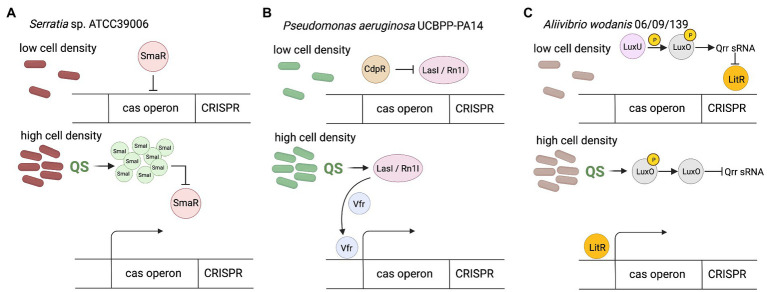
CRISPR-Cas regulation by quorum sensing. **(A)** In *Serratia* sp. at low cell density in the absence of SmaI autoinducer SmaR acts as the repressor of the expression of *cas* genes and CRISPR array. The increase of cell density lead to SmaI accumulation, which bind to SmaR, thereby inhibiting its DNA binding activity and resulting in derepression of expression of CRISPR-Cas system. **(B)** In *Pseudomonas aeruginosa* at low cell density CdpR indirectly inhibits binding of virulence factor regulator (Vfr) to the cas promoter by repressing QS regulators (LasI/RhlI). Increase of cell density causes that LasI/RhlI activate expression of the *cas* operon by stimulating Vfr binding. Vfr is required for the CdpR-mediated regulation of CRISPR-Cas function. The function of Vfr is activated by QS autoinducers and repressed by QS inhibitors, so depends on cell density (CdpR represses QS regulators to inhibit CRISPR-Cas immunity through the Vfr signaling. However, the detailed mechanism remains to be defined). **(C)** In *Aliivibrio wodanis* at low cell density, when the AIs concentration is low, the receptors LuxU relays phosphate to LuxO, which activates the expression of Qrr sRNA. The Qrr sRNA inhibits the expression of LitR. At high cell density dephosphorylate LuxO occurs and inactivation of Qrr sRNA. Inactivation of Qrr sRNA activates LitR, positively regulates expression of the the *cas* operon. Created with BioRender.com

Certain groups of bacteria regulate their behavior according to density of cells through the quorum sensing (QS) system (Miller and Bassler, 2001). The presence of many signaling molecules (autoinducers) produced by bacteria provides feedback about increased cells density. In general, Gram-negative bacteria use acylated homoserine lactones (AHLs) as autoinducers, whereas Gram-positive bacteria use processed oligopeptides to communicate (Miller and Bassler, 2001). After exceeding the threshold concentration of the autoinducer in the environment, changes in gene expression within the entire population occur. It is a widespread form of communication, necessary to confer advantageous traits in a bacterial community, living in dense aggregation, which makes them vulnerable to temperate viral infections (Knowles et al., 2016) and horizontal gene transfer (Pinedo and Smets, 2005). In relation to CRISPR-Cas regulation, the studies of Gram-negative genus *Serratia* have shown that both the adaptive and interference phases of subtypes: I-E, I-F and III-A CRISPR-Cas systems are regulated by quorum sensing (Patterson et al., 2016). It was observed that the presence of AHL autoinducers in the environment improves the generation of immune memory in high-density populations by promoting increased acquisition of new spacers, however the exact mechanism of this phenomenon remains unknown. Generally, QS in Gram-negative species employs LuxI family proteins to generate N-acyl homoserine lactone AHLs, which are sensed by LuxR-type transcriptional regulators. Yet, studies of *Serratia* performed by Patterson et al. (2016) revealed that SmaR which is LuxR homolog is involved in this process. In the absence of SmaI autoinducer molecules in the environment SmaR acts as the repressor of the expression of *cas* genes and CRISPR array. However, the increase of cell density led to AHLs accumulation, which in turn bind to SmaR, thereby inhibiting its DNA binding activity and resulting in derepression of expression of CRISPR-Cas system (Figure 1A). This observation indicates that bacteria living in dense populations, and thus more susceptible to viral infections, can sense the increased concentration of the autoinducer, which leads to increased acquisition of new spacers through the expression of genes involved in this process (*cas* genes and crRNA-encoding genes). A correlation of QS with the regulation of the CRISPR-Cas system by autoinducer molecules has also been observed in *Pseudomonas aeruginosa* (Høyland-Kroghsbo et al., 2017).

The CdpR (ClpAP-degradation and pathogenicity Regulator) protein from the AraC-family transcriptional factors can interact with the ClpAS-P-ClpP protease to negatively modulates the expression of virulence factors in *P. aeruginosa*, but also has been proven to be an important QS regulator (Zhao et al., 2016). CdpR directly regulates many genes, but it can also play a role in gene regulation by interacting with other proteins or small molecules. The CdpR binds directly to the promoter region of pqsH gene which encodes the key synthase of *Pseudomonas* quinolone signal (PQS) and regulates its expression. Recently, the mechanism for the regulation of CRISPR-Cas defense systems by the CdpR was described (Lin et al., 2019). CdpR indirectly inhibits binding of virulence factor regulator (Vfr) to the *cas1* promoter by repressing QS regulators, while LasI/RhlI autoinducers activate expression of the *cas* operon by stimulating Vfr binding (Figure 1B). Decreased *cas1* expression in Δvfr strain weakens the defense response of the CRISPR-Cas system causing a more severe course of phage infection, and on the other hand, causes limitation of the cleavage of endogenous bacterial sequences driven by Cas3 RNA reducing in this way self-targeting activity of the CRISPR-Cas system. This would be an additional safety mechanism that can support the PAM-based safety lock. Therefore, CdpR regulation is another layer of organization for bacterial antiphage intracellular signaling, furthermore involved in ensuring homeostasis of the cell (Lin et al., 2019).

A similar dependence has been observed in *Aliivibrio wodanis* 06/09/139, a bacterium that has two QS systems, the LuxS/LuxPQ master system and the AinS/AinR system *via* N-acyl-homoserine (AHL), and the master QS regulator, LitR (Maharajan et al., 2022). At low cell density the receptors (AinR and LuxPQ) can act as kinases and relay phosphate to LuxO *via* LuxU, which activates the expression of *qrr* sRNA (Figure 1C). The Qrr sRNA inhibits the expression of *litR*. When the autoinducers concentration is high, at high cell density, they bind to the receptors to dephosphorylate LuxO and inactivate *qrr* sRNA. Inactivation of *qrr* sRNA, in turn, activates *litR*. Therefore, the research has shown that LitR regulates CRISPR system in a cell density manner - the expression of *cas* genes (cas1, cas3, and csy3) was increased at high cell density compared to low cell density. This suggests that *A. wodanis* positively influences *cas* genes as the cell density increases, which may provide protection upon phage infection at higher cell density.

While the available data on correlation of QS with the regulation of the CRISPR-Cas system are currently limited, the broad distribution of both CRISPR-Cas and QS systems within diverse bacteria suggests that QS-dependent regulation of immunity would be widespread. This assumption is further supported by the fact that the increased frequency of acquiring new spacers makes the bacteria better adapted to the environment and increases the chances of their survival in dense populations.

### 2.2. Nucleoid-associated proteins

Nucleoid associated proteins (NAPs) have the ability to bind double stranded DNA. In addition to their structural functions, i.e., the ability to organize DNA into higher order structures, they act as global regulators of gene expression (Dillon and Dorman, 2010). The function related to the CRISPR-Cas system regulation has been reported for proteins belonging to the NAPs family, namely histone-like nucleoid structuring (H-NS) protein, DNA-binding protein StpA and LRP. H-NS proteins bind to DNA in a non-specific manner, in regions rich in adenine and thymine, leading to a strong condensation of DNA, which in turn may inhibit gene transcription resulting from inaccessibility of promoter sequences for the sigma subunit of RNA polymerase (Navarre et al., 2007; Stoebel et al., 2008). The studies have shown that H-NSs can also down regulate the transcription of the CRISPR-Cas genes. It was found that in *Escherichia coli cas* genes expression under laboratory conditions is almost completely repressed by H-NS activity (Pul et al., 2010). Furthermore, some phages were shown to encode their own *hns* genes, which leads to the hypothesis that they could suppress the defense response of the CRISPR-Cas system (Skennerton et al., 2011). Expression of *cas* genes therefore requires mechanisms to counteract phages directed silencing and to relieve the repression caused by H-NS. The transcription of CRISPR array also depends, although to a lesser extent, on the regulatory properties of these proteins (Pul et al., 2010). It is due to the lower binding affinity of H-NS to the leader part of the CRISPR array which allow generation pre-crRNA at low levels as the expression is not completely turned off (Pul et al., 2010).

**Figure 2:**
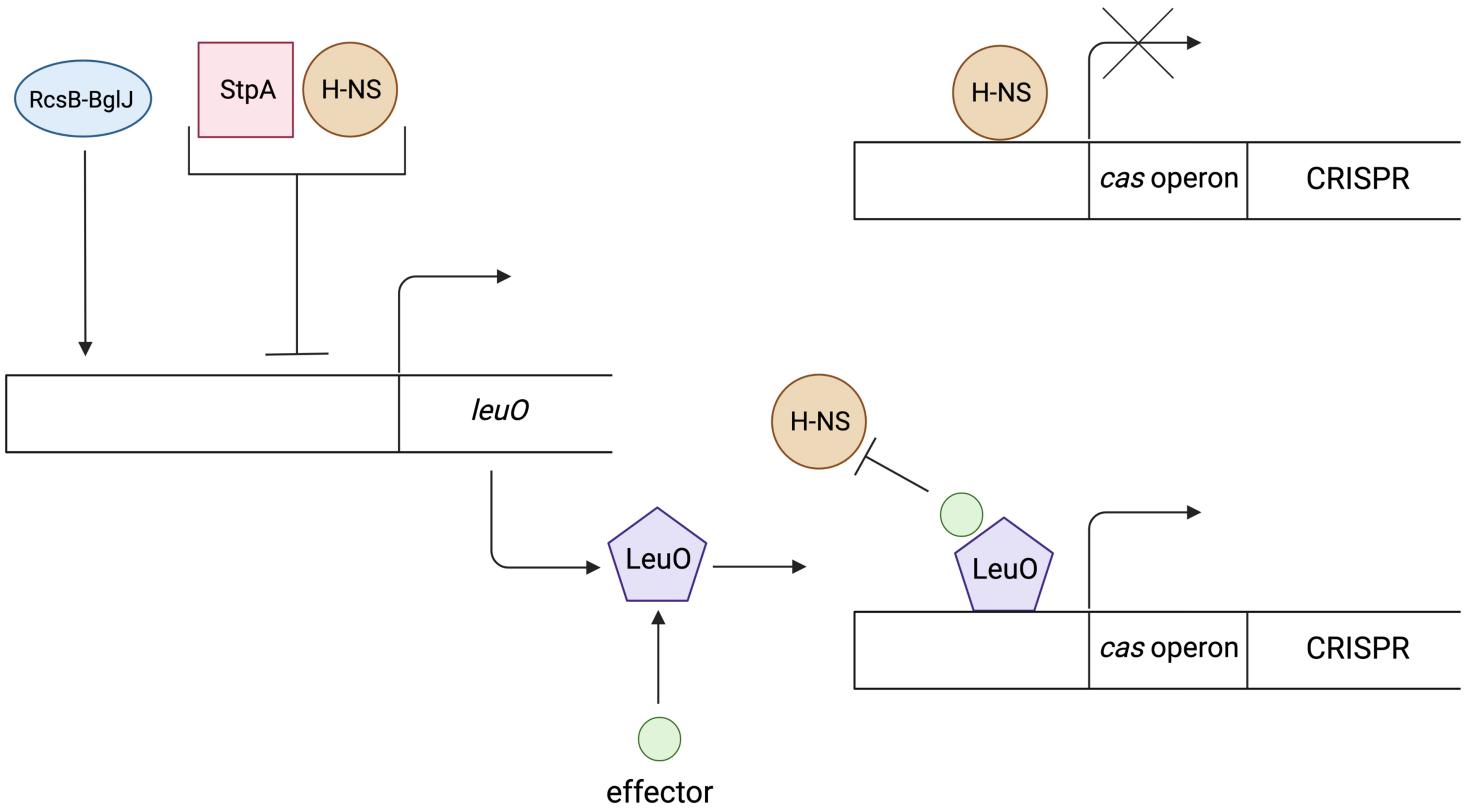
The mechanism of regulation and action of the LeuO protein. Expression of the LeuO proteins is inhibited by the H-NS, StpA proteins and by the LeuO protein (after reaching a high level in a cell). Expression is activated *via* the RcsB-BglJ heterodimer. The LeuO protein requires a specific signal (effector) to counteract H-NS-mediated repression of *cas* gene transcription. Created with BioRender.com.

Another protein that is worth mentioning is LeuO. Although it does not belong to the NAPs family, it is the best-known antagonist protein for the H-NS. The LeuO belongs to the family of transcription factors named LysR-type transcriptional regulator (LTTR). LTTRs contain two pairs of DNA-binding domains (DBD) and two pairs of effector-binding domains (EBD). The regulatory protein LeuO binds to DNA in the regulation sequence of *cas* gene expression as a tetramer, which interferes with the cooperative binding of H-NS proteins (Guadarrama et al., 2014). Specific effector that promotes transcription of *cas* genes by LeuO remains unknown, therefore research is being carried out using plasmid encoded *leuO* under control of constitutive or inducible promoters (Fragel et al., 2019). Studies on *E. coli* mutants showed that the level of transcription of the *cas* operon was higher in cells with inducible expression of the *leuO* gene than in cells lacking genes encoding H-NS repressor proteins, suggesting that LeuO not only prevents repressor binding but also promotes transcription of these genes or that derepression in K12Δ*hns* is incomplete (Westra et al., 2010). Expression of the *leuO* genes is also regulated which indirectly also influences the regulation of the CRISPR-Cas system. Expression is activated *via* the RcsB-BglJ heterodimer (Venkatesh et al., 2010). Both RcsB and BglJ belong to the LuxR family of transcription regulators. RcsB is a regulator of a two component system that detects disturbances in the outer membrane and peptidoglycan (Majdalani and Gottesman, 2005; Kleinstiver et al., 2016). that can occur during phage infections. Repression of *leuO* transcription is caused by H-NS and/or StpA (H-NS homolog). Moreover, a negative feedback loop is observed in the case of high LeuO concentration. LeuO protein can bind to DNA in its own operator and inhibit transcription (autorepression; Stratmann et al., 2012). Figure 2 shows the regulation mechanism of the LeuO protein.

Another protein belonging to NAPs family is StpA. Recent studies indicate a dual function of the StpA protein being a homologue of the H-NS protein. It turns out that this protein may influence the expression of *cas* operon. When StpA is transcribed at low level it acts opposite to its paralog – the H-NS. Furthermore, the discovery that the StpA and H-NS paralogs share a common DNA binding site but have opposite roles in the regulation of transcription indicates that chromatin condensation by histone-like proteins may act in opposition to the transcription process (Sun et al., 2019). Other studies considering high level StpA transcription show that this represses the transcription of *cas* genes of the I-E subtype in *E. coli* (Mitić et al., 2020).

Leucine-responsive regulatory proteins (LRPs) also belong to the NAPs family and have a similar function to the H-NS proteins. However, they have no effect on the I-E subtype in *E. coli* and are repressive on the I-E subtype in *Salmonella enterica*. Unlike most NAP proteins which do not exhibit binding specificity, LRP binds to a specific nucleotide sequence (Herz and Strickland, 2001). It can be concluded that the CRISPR-Cas regulation evolved independently in different strains and most likely reflects the differences in selective pressures resulting from the presence of different infections (Medina-Aparicio et al., 2011).

### 2.3. cAMP receptor protein

cAMP receptor proteins are also called catabolite activator proteins (CAP). The cAMP-CAP complex is part of the global transcription modulon, positively controlling a group of operons responsible for the catabolism of various sugars. Catabolic repression is observed when in the absence of glucose and in the presence of another sugar, the level of cAMP synthesized by adenylate cyclase from ATP increases. CAP protein, which is a positive regulator of other than glucose catabolic operons, binds to its target site only in the presence of cAMP. To modulate gene expression, the CAP-cAMP complex must bind to a specific sequence within the promoter region. This complex supports transcription by stimulating binding of RNA polymerase to the promoter and by increasing its processivity at low glucose levels in the cell (Perlman et al., 1969; Hahn et al., 1984; Reznikoff, 1992; Kolb et al., 1993; Ishizuka et al., 1994). The role of the CAP proteins has also been described in the context of the regulation of the CRISPR-Cas system. It was shown that the cAMP-CAP complex exerts different regulatory effects on expression of CRISPR-Cas systems in different bacteria species. In one case, glucose deficiency activates *cas* gene expression in *Pectobacterium atrosepticum* (CRISPR-Cas subtype I-F) but the opposite regulatory effect is seen at high glucose levels (Patterson et al., 2015), while in *E. coli* (subtype I-E) elevated glucose levels lead to indirect activation of transcription and the opposite glucose levels lead to inhibition of transcription (Yang et al., 2014). Different effect in *E. coli* and *P. atrosepticum* is due to the presence of CAP binding sites at various locations in the genome. Analysis of the CRISPR-Cas regulatory elements in *E. coli* revealed a CAP binding site, which is located between −281 and − 259 bp upstream of the *cse1* transcriptional start site and overlaps the binding site of the LeuO activator. The *cse1* operon contains 7 genes (*cse1*, *cse2*, *ces4*, *cas5e*, *cse3*, *cas1*, and *cas2*) so the regulation of *cse1* promoter has a direct effect on expression of *cas* genes and CRISPR array. On the contrary, *P. atrosepticum* CAP-box is positioned optimally (centered at −41.5 bp) adjacent to the −35 site of the *cas1* promoter which allows the enhancing RNAP binding and has no effect on the *leuO* binding site. The *cas1* promoter drives expression of the entire *cas* operon so upregulation of *cas* expression within this subtype correlates with increased interference and adaptation.

**Figure 3:**
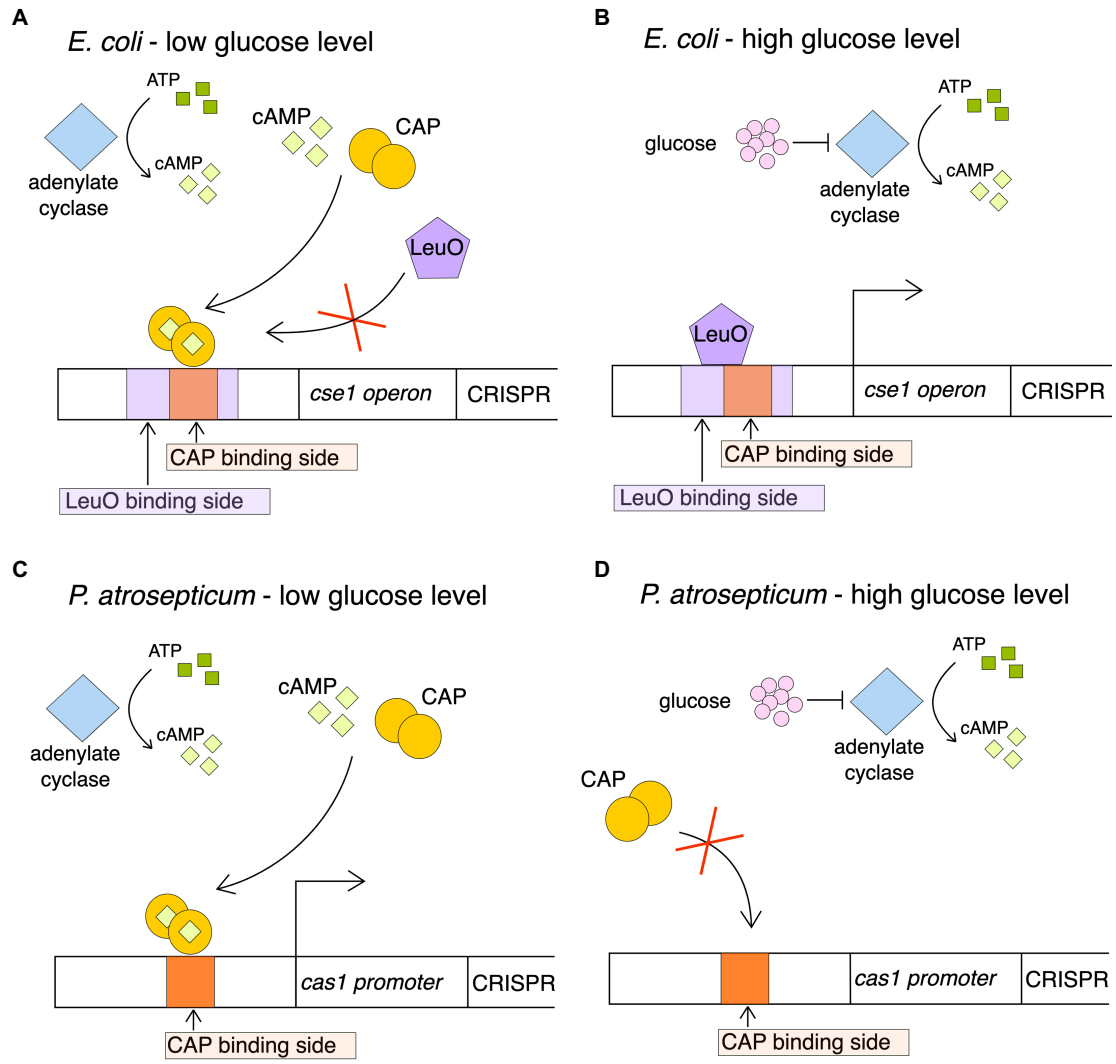
The regulation mechanism of cAMP-CAP in *E. coli* in a low glucose concentration **(A)**, transcription of the *cas* genes and CRISPR array is repressed by binding of the cAMP-CAP complex. Binding of the complex prevents binding of the protein LeuO, which is a positive transcription regulator. On the other hand, increasing concentration of glucose **(B)** results in a decrease in the number of active complexes, which cannot compete with LeuO in binding to the LeuO binding side. The bound LeuO protein activates the transcription of the cas genes and the CRISPR RNA precursor (pre-crRNA). The regulation mechanism of cAMP-CAP in *P. atrosepticum* in a low glucose concentration **(C)**, low glucose concentration results in the activation of adenylate cyclase, which is involved in the conversion of ATP to cAMP. CAP, which is a positive regulator of the *cas* operon, binds to its target sequence only in the presence of cAMP. The cAMP-CAP complex binds to the *cas* gene promoter and activates the *cas* operon. High glucose concentration **(D)**, transcription of cas genes is repressed by the glucose phosphotransferase system and more specifically by adenylate cyclase. The lack of cyclase means that ATP is not converted to cAMP, and thus the active cAMP-CAP complex is not formed, which negatively regulates the transcription of *cas1* promoter.

*P. atrosepticum* regulation mechanism is shown in Figure 3. When glucose concentration reaches threshold level, the expression of CRISPR-Cas I-F subtype system is limited by the repression of adenylate cyclase, thus preventing unnecessary resource and energy costs generated by expression of the *cas* operon. Such control resembles catabolic repression. Acquisition and replication of extra-chromosomal elements such as phages, transposons and plasmids can disrupt stable metabolic pathways. Signals resulting from a lack of nutrients are detected by the increased production of cAMP. Thereby, the amount of the cAMP-CAP complex in the cell increases, which promotes the induction of a targeted response against exogenous DNA. CAP-cAMP binds to the *cas* promoter and activates the operon of the *cas* genes encoding Cas proteins involved in both adaptation and interference.

*E. coli* regulation mechanism is also shown in Figure 3. The binding region of the CAP-cAMP complex and the LeuO protein overlap. When glucose level is high, low cAMP level limits competition of the CAP-cAMP complex with the LeuO activator, which promotes *cas* expression. In this situation, the *cas* genes and the CRISPR region can only be expressed when the level of cAMP is too low for CAP. This happens when the glucose concentration inside the cell decreases. This regulatory system reflects the ability of *E. coli* to strategically allocate limited energy between growth and immune responses. The bacterium, in the absence of energy sources, switches off the systems that are not currently necessary for its survival. Recent studies highlight that the metabolic state of the host cell is associated with the induction of CRISPR-Cas immunity (Patterson et al., 2015). What is more, *cas3* expression is also regulated by CAP in *E. coli* (Yang et al., 2020). This mechanism is closely linked to the GCS, which is also known as the glycine decarboxylase complex (GDC). This system is a series of mitochondrial enzymes that are triggered in response to high concentrations of the amino acid glycine.

The differences of the cAMP-CAP complex activity on the regulation of the CRISPR-Cas system may be explained by the different ecological niches of both microorganisms from the *Enterobacteriaceae* family. *E. coli* is often found in bacterial flora of the large intestine of animals and *P. atrosepticum* is a plant pathogen. Differences in nutrient availability may explain why the same stimulus regulates CRISPR-Cas expression differently. This allows to conclude that the control of the CRISPR-Cas system is niche specific. It should be remembered that not all acquired mobile genetic elements are harmful, on the contrary, they may benefit prokaryotes, e.g., through the presence of antibiotic resistance genes, genes determining adaptive traits or the presence of other genes that may be beneficial, and under some conditions necessary for survival. Cells with active CRISPR-Cas systems could be killed and bacteria capable of maintaining a downregulated level of CRISPR-Cas gene expression would survive because the beneficial genes would not be degraded (Jiang et al., 2013). Thus, it has been proposed that this type of regulation also has the effect of limiting the removal of beneficial mobile elements. Adverse bacteriophage infections require both CAP and cAMP to break free from the lysogenic cycle and enter the lytic cycle (Friedman et al., 1984; Osterhout et al., 2007; Maynard et al., 2010a, 2010b). Therefore, they use the same factors that stimulate the expression of the CRISPR-Cas system genes. It is possible that these systems operate in a way of incomplete protection (reduced expression of CRISPR-Cas), which may first allow entry of a plasmid or phage, which will only be preserved if they turn out to be beneficial to the host (Patterson et al., 2015).

### 2.4. G protein

High temperature protein G (HtpG) is present in various bacteria including *E. coli.* Like many chaperones, it is responsible for ensuring the correct conformational structure and the right cellular localization of newly synthesized polypeptides. It also participates in the reactivation or degradation of proteins damaged by, e.g., heat shock, and therefore its level increases sharply at elevated temperatures (Thomas and Baneyx, 2000). Its role as a positive modulator of the CRISPR-Cas system has been discovered relatively recently (Yosef et al., 2011). HtpG stabilizes the Cas3 protein, the key component of the interference phase (Majsec et al., 2016). Studies have shown that HtpG proteins present in *E. coli* mutants lacking in the *hns* genes (which code H-NS proteins that are repressors of Cas protein expression) are important for the activity of CRISPR-Cas systems at 30 and 32°C (Yosef et al., 2011). The presence of functional Cas3 proteins in cells at higher temperature (37°C) decreases, and thus the resistance of bacteria under these conditions is much lower than at 30 and 32°C. Phage resistance can be restored at 37°C, to a level comparable to that at 30 and 32°C, by inducible *cas3* expression from plasmids (Majsec et al., 2016). *In silico* studies did not show a correlation of the coexistence of both proteins – Cas3 and HtpG in different strains (Majsec et al., 2016). It is likely that different organisms have other proteins that interact with Cas3.

### 2.5. VicR/VicK

*Streptococcus mutans* has two CRISPR-Cas systems in its genome classified as subtypes II-A and I-C. Their expression is regulated by the two component VicR/K system. In prokaryotes, two-component signaling systems (TCSs) usually consist of a histidine kinase (HK), and a response regulator (RR), here VicK and VicR, respectively. The regulatory relationship of the VicR/K and CRISPR-Cas system has been described so far only once (Serbanescu et al., 2015). Transcriptional analysis revealed that the VicR/K signal transduction system increased the expression of subtype II-A *cas* genes, while the level of Cas proteins of subtype I-C was reduced. So far, the mechanism how the two-component system influences the control of CRISPR-Cas activity is unclear. Yet, its regulatory role has also been observed in the context of biofilm formation, bacterial competence, response to oxidative stress (Deng et al., 2007) or stress related to cell membrane disruption (Duque et al., 2011).

### 2.6. KinB/AlgB

The two-component KinB/AlgB system has been characterized as the regulation system of alginate biosynthesis in *Pseudomonas aeruginosa* (Damron et al., 2009, 2012). Alginate is an extracellular polysaccharide, overproduced by bacterial species found in the lungs of people with cystic fibrosis (Bjarnsholt et al., 2009). *P. aeruginosa* has an active CRISPR-Cas system of the I-F subtype, the repression of which is observed with an increase in the level of the phosphorylated form of the AlgB protein, which activates the repressors: AlgU, AlgR and AmrZ. The dephosphorylation of AlgB is enabled by the KinB protein (Borges et al., 2020). The same endogenous repression pathway of CRISPR-Cas activity also induces alginate production and bacterial surface association. The decreased activity of the immune system can therefore be justified, since biofilm formation reduces the risk of phage infection (Heilmann et al., 2010). This is an example of strategy minimizing energy consumption. Mobile genetic elements that target *Pseudomonas* bacteria have AmrZ homologues that suppress expression and activity of CRISPR-Cas system (Borges et al., 2020).

### 2.7. Csa3 and Cascade

**Figure 4:**
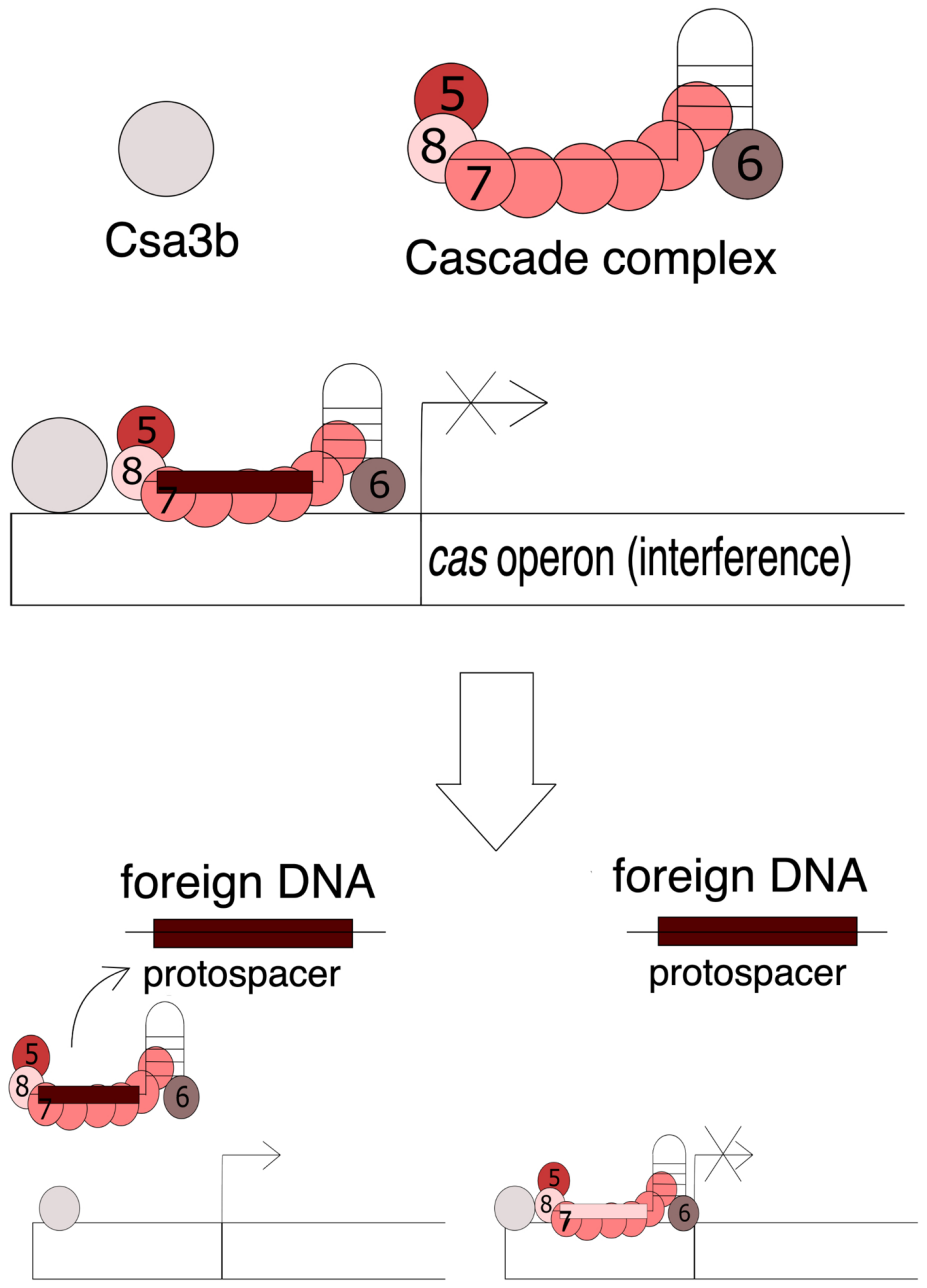
Mechanism of the Cas3b-Cascade action. The attachment of the Cascade complex is dependent on the Cas3b protein. Both Cas3b and Cascade bind to the promoter region of the *cas* genes encoding proteins involved in the interference phase, resulting in the inhibition of expression of these genes. Presence of foreign DNA, e.g., viral DNA, containing the complementary protospacer sequence causes the detachment of the complex and activation of gene transcription. Numbers 5, 6, 7, and 8 stand for Cas5, Cas6, Cas7, and Cas8 proteins, respectively.

The adaptive phase is essential for the CRISPR-Cas system to be functional. Recruitment of new spacers requires the participation of the Cas1 and Cas2 proteins, which in some CRISPR-Cas types are regulated by the Csa3 protein. The *Sulfolobus islandicus* genome encodes two variants of Csa3: Csa3a and Csa3b. Overexpression of the Csa3a protein significantly increases the transcription efficiency of *cas1* and *cas2* genes, which results in an increased ability to acquire new spacers (Liu et al., 2015). Moreover, increased levels of Cas1 and Cas2 proteins result in obtaining spacers encoded in the vicinity of a less conserved PAM sequences. It has been demonstrated that Csa3a protein co-activates multiple repair genes, including herA helicase, nurA nuclease and DNA polymerase II genes, which are essential during the adaptation stage when repair systems restore the integrity of the CRISPR array after spacer insertion (Liu et al., 2015; Faure et al., 2019). The function of the Csa3b protein differs significantly from the Csa3a protein, as it regulates the activity of the gene cassette encoding proteins involved in the interference phase of the archaea *Sulfolobus solfataricus* – Cas1 and Cas2. On default, the Csa3b protein, together with the crRNA-bound Cascade complex, binds in the promoter region and maintains transcriptional repression (He et al., 2017). Binding of Cascade to the promoter region of genes involved in CRISPR-Cas interference depends on the Csa3b protein. Both Cas3b and Cascade bind to the promoter region of the *cas* genes, resulting in the inhibition of expression of these genes. In the course of infection, the Cascade complex detaches itself in search of protospacers encoded in foreign, invasive genetic material. Figure 4 shows this mechanism of action. Taken together, these studies showed that the Cascade complex with the Csa3b protein repressed the expression of interference proteins. Upon cellular entry of exogenous DNA containing the PAM motif, the Cascade complex disengages, activating the expression of genes encoding Cas proteins involved in the interference phase, which enables a quick reaction to the threat (Li et al., 2016).

### 2.8. Anti-CRISPR proteins

Like any defense system, CRISPR-Cas is involved in a constant “arms race” with phages, which causes the rapid evolution of both CRISPR-Cas systems and phages (Takeuchi et al., 2012) causing a significant diversity of genes and the structure of the CRISPR-Cas locus. This is mostly because of relationship between a prokaryote and phage and their continual adaptation and counteradaptation of defense and attack strategies (Westra et al., 2016). Phage evolution is mainly based on minimizing the detection of infection by the protective systems of bacteria. A key feature of the CRISPR-Cas systems is their strict specificity of action that phages can use against bacteria. Mutations in bacteriophage genetic material, which are usually found in PAM sequences or in protospacer regions often referred to as “escape mutations,” may lead to ineffective operation of the Cas protein machinery as the exogenous DNA becomes more difficult to detect or not detected at all (Deveau et al., 2008; Semenova et al., 2011). However, the efficacy of such mutations may not be sufficient in a bacterial population with a large variety of spacers. Some phages and other mobile genetic elements have developed refined strategies to evade the CRISPR-Cas immune system, producing small (50 to 150 amino acids) Anti-CRISPR (Acr) proteins which inhibit the interference machinery directly. To date, over 300 Acr proteins have been identified (Wang et al., 2021). These can be divided based on inhibited CRISPR types, stages of inhibition, and the Cas proteins they target. The most common mechanisms of Acr-based CRISPR-Cas systems were listed in Table 1. The *acr* genes are often found closely together in the genome of viruses and bacteria, where they found their way through mobile genetic elements. This allowed researchers to discover them. Many algorithms are currently in use to facilitate this process (Eitzinger et al., 2020; Gussow et al., 2020; Wang et al., 2020). The Acr proteins are named after the system that they inhibit in the order they were discovered (Bondy-Denomy et al., 2018). For example, the AcrIIA4 protein was the fourth discovered Acr, which inhibits CRISPR-Cas system subtype II-A.

**Table 1:** Anti-CRISPR proteins mechanism of action. Adapted from Marino et al. (2020) and Wang et al. (2021).

Inhibited process	Acr protein	Mechanism of action
Target DNA binding	AcrIF1	binds to the Csy complex (to Cas7 protein; Pawluk et al., 2016; Bondy-Denomy et al., 2018)
	AcrIF2	binds to the Cascade complex (to Cas8 and Cas7 proteins; Chowdhury et al., 2017)
	AcrIF4	binds to the Cascade complex but the exact binding locations is unknown (Bondy-Denomy et al., 2015; Pawluk et al., 2016)
	AcrIF9	binds to the Cascade complex and induces system to bind dsDNA independent of sequence complementarity or PAM (Hirschi et al., 2020; Kim et al., 2020; Lu et al., 2021)
	AcrIF10	binds to the Cascade complex (to Cas8 and Cas5 proteins; Pawluk et al., 2016; Guo et al., 2017)
	AcrID1	binds to the Cascade complex (to Cas10; He et al., 2018)
	AcrIIA2	binds to the Cas9 endonuclease but the exact binding locations is unknown (Rauch et al., 2017; Bondy-Denomy et al., 2018)
	AcrIIA4	mimics double-stranded DNA, binds to the Cas9-sgRNA complex (Shin et al., 2017; Yang and Patel, 2017)
	AcrIIA6	acts as an allosteric inhibitor and induces Cas9 dimerization (Hynes et al., 2018; Fuchsbauer et al., 2019; Zhang et al., 2019)
	AcrVA1	cuts off the 3′ end of the crRNA (Knott et al., 2019; Zhang et al., 2019)
	AcrVA4	dimerizes the Cas12a-crRNA complex (Knott et al., 2019)
	AcrVA5	acylates the Cas12 lysine residue that interacts with the PAM motif (Watters et al., 2018; Dong et al., 2019)
	AcrIIC3	dimerizes the Cas9 endonuclease (Sun et al., 2019)
	AcrIIC4	interacts with Cas9 but the exact mechanism is unknown (Lee et al., 2018)
	AcrIIC5	interacts with Cas9 but the exact mechanism is unknown (Lee et al., 2018)
Target DNA cleavage	AcrIIC2	interactions with the positively charged bridge helix of Cas9 endonuclease, thereby preventing sgRNA loading (Thavalingam et al., 2019; Zhu et al., 2019)
	AcrIIC1	binds to the HNH domain of the Cas9 endonuclease (Zhu et al., 2019)
	AcrIIA11	interacts with Cas9 and dsDNA but the exact mechanism is unknown (does not prevent target recognition; Forsberg et al., 2019; del Banco et al., 2020)
	AcrIE1	binds to Cas3 and blocks Cas3 recruitment to Cascade complex (Pawluk et al., 2017)
	AcrIF3	binds to Cas3 and blocks Cas3 recruitment to Cascade complex (Bondy-Denomy et al., 2013, 2015)
Csx1 RNase*	AcrIIIB1	binds Cmr effector complexes, probably blocks Csx1-activating signal (Bhoobalan-Chitty et al., 2019)

* CRISPR-Cas III requires the participation of the Csx1 protein, which has RNase activity activated by Co-A, in the interference phase. Viruses encoding Acr proteins that have a repressive effect on DNase do not survive, because the accumulation of Co-A will cause death of the bacterial cell, i.e., its host (Bhoobalan-Chitty et al., 2019).

The functions of Acr proteins known so far are very diverse and can be divided into two main modes of action. The first is based on the inhibition of target DNA binding, and the second type of mechanism is DNA cleavage inhibitory proteins. Each one of them enables foreign genetic material to avoid degradation by CRISPR-Cas systems. Some Acr proteins are highly subtype specific (Bondy-Denomy et al., 2013), while others have a broader spectrum of activity (Pawluk et al., 2016).

**Figure 5:**
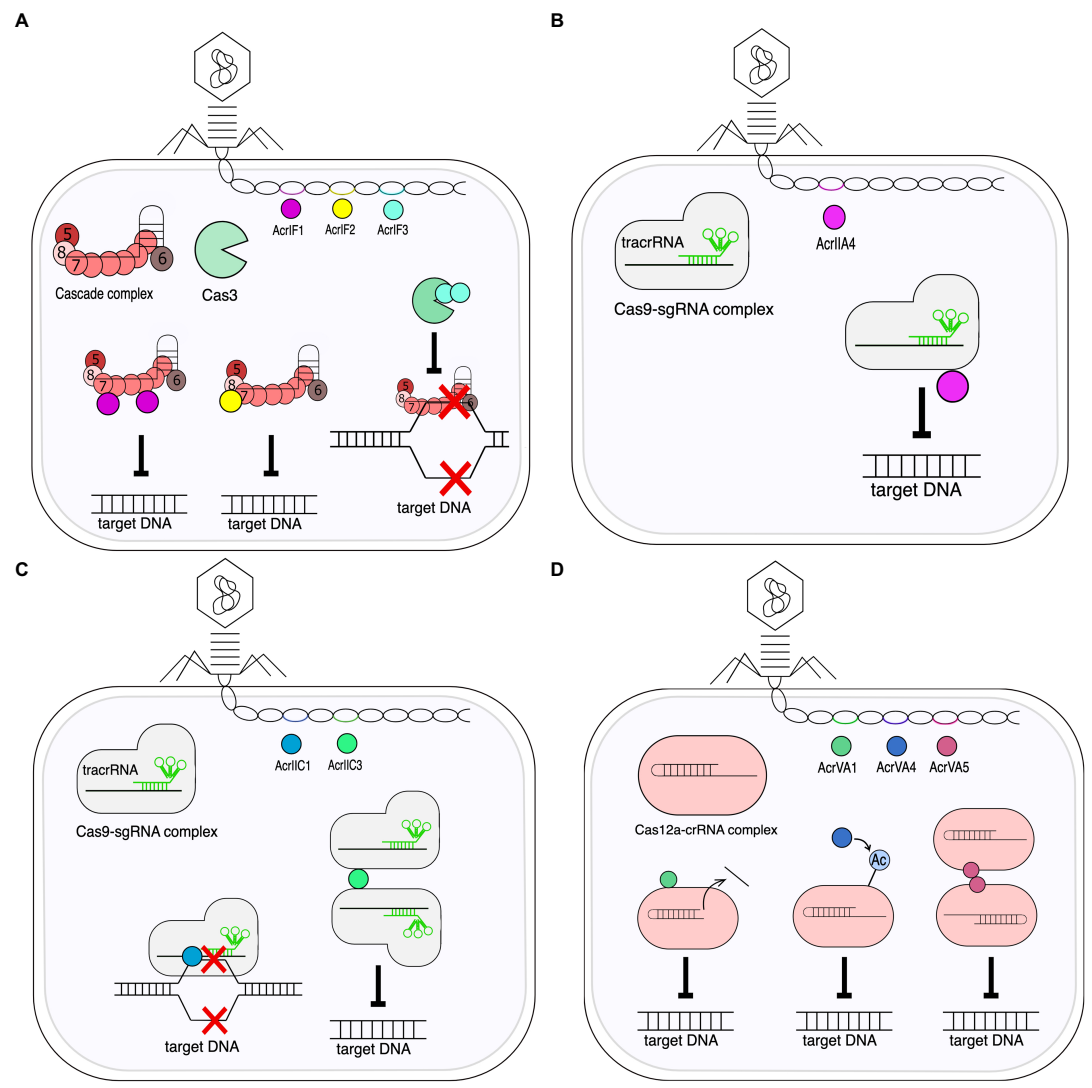
Mechanisms of action of Acr proteins. **(A)** Mechanism of AcrVA1, AcrVA4, AcrVA5. AcrVA4 and AcrVA5 inhibit double stranded DNA recognition. AcrVA4 dimerizes the Cas12a-crRNA complexes, and the AcrVA5 protein acylates a Cas12 lysine residue that interacts with the PAM motif. AcrVA1 cuts the 3′ end of the crRNA and thus prevents recognition of the protospacer. **(B)** Mechanism of AcrIIA4. The AcrIIA4 protein mimics double stranded DNA and blocks target DNA recognition by binding to the Cas9-sgRNA complex. **(C)** Mechanism of AcrIIC1 and AcrIIC3. The AcrIIC1 protein binds to the HNH domain of the Cas9 endonuclease, thereby disrupting its nucleolytic activity and preventing cleavage target DNA. The AcrIIC3 protein, on the other hand, blocks binding to the target DNA by inducing Cas9 dimerization. **(D)** Mechanism of AcrIF1, AcrIF2, and AcrIF3. AcrIF1 by attaching to the Cascade complex (Cas7) inhibits binding to the target DNA. By binding to the complex at different sites (Cas8 and Cas7), the AcrIF2 protein also blocks binding to exogenous DNA. In contrast, the AcrIF3 protein binds to Cas3 nuclease and prevents cleavage the target DNA.

Research on *P. aeruginosa* revealed one of the action mechanisms of Acr proteins (Bondy-Denomy et al., 2013). AcrIF1 and AcrIF2 have been shown to directly interact with the I - F effector complex, inhibiting its ability to bind DNA. While the action of both Acr proteins has the same effect, they interact with different subunits of the effector complex. Two to three copies of the monomeric AcrIF1 protein bind to the Cas7 hexamer which forms the backbone of the Cascade. This leads to conformation changes and exposure of lysine residues, blocking access to target DNA binding. In contrast, the AcrIF2 protein binds to Cas8f between Cas7f backbone tail end of the Cascade. This in turn prevents DNA binding by competing with the target sequence for critical interaction with the two positively charged helices on the adjacent Cas7. In turn, AcrIF3 binds as a dimer to Cas3 and keeps it bound to ADP, thereby preventing its recruitment into the Cascade complex, blocking the digestion of the target DNA. The action mechanism of AcrIF1, AcrIF2, AcrIF3 is shown in Figure 5A.

AcrIIA4 protein mimics double stranded DNA and blocks target DNA recognition through a number of mechanisms: competitive binding of the PAM motif, inhibition of DNA coiling and loop structure formation, blocking HNH (His-Asn-His) domain movements necessary to catalyze complex attachment reactions. Moreover, AcrIIA4 only binds to Cas9 in the presence of sgRNA in a 1:1 ratio. The Cas9-sgRNA complex associated with AcrIIA4 shows a similar proteolytic digestion pattern as the Cas9-sgRNA complex itself, suggesting that AcrIIA4 binding does not alter the Cas9-sgRNA conformation (Dong et al., 2017; Shin et al., 2017; Yang and Patel, 2017). The mechanism of AcrIIA4 inhibition is shown in Figure 5B.

The AcrIIC1 protein, which inhibits type II-C systems, binds the HNH domain of the Cas9 endonuclease. Binding to the target sequence is retained, but the necessary conformational changes required for the Cas9 protein to function as an active nuclease are hampered. The AcrIIC3 protein, on the other hand, blocks binding to target DNA by inducing Cas9 dimerization (Harrington et al., 2017). The mechanism of action of AcrIIC1 and AcrIIC3 is shown in Figure 5C.

Inhibition of bacterial Cas12a nuclease activity by AcrVA1, AcrVA4, and AcrVA5 occurs through functionally distinct mechanisms. The AcrVA4 and AcrVA5 proteins inhibit the recognition of double-stranded DNA (dsDNA). AcrVA4 dimerizes Cas12a-crRNA complexes (Knott et al., 2019). The AcrVA5 protein, independently of the crRNA, acylates the Cas12 lysine residue interacting with the PAM motif, which causes the loss of the hydrogen interaction between two domains constituting the Cas protein, as well as steric obstacle, thus preventing the recognition of the PAM motif in dsDNA (Watters et al., 2018; Dong et al., 2019). In contrast, AcrVA1 cuts off the 3′ end of the crRNA, thus making it impossible to recognize the protospacer (Knott et al., 2019). The mechanism of AcrVA1, AcrVA4, and AcrVA5 is shown in Figure 5D.

Additionally, another CRISPR-Cas9 inhibitory protein was discovered, that does not belong to the Acr group. It is the peptide derived from the periplasmic domain of inoviridae bacteriophages (including commonly used laboratory bacteriophage strain M13) major coat protein G8P (G8PPD; Cui Y. R. et al., 2020). G8PPD inhibits Cas9 activity by disrupting Cas9 and sgRNA binding in an allosteric manner, rather than competing directly with sgRNA loading. G8PPD can only show inhibitory effects when G8PPD is overexpressed before sgRNA transfection, otherwise, inhibition is reduced and may prevent excess Cas9 from off-target editing.

## 3. General natural patterns of the CRISPR-CAS regulation

CRISPR-Cas systems include multiple elements, which makes them metabolically expensive. What is more, due to their nature they can pose a threat to the host cell if left unchecked. Therefore, the systems are activated only under specific external and internal conditions. This is probably because cells try to balance between efficient protection and reducing its cost. Such balancing can be observed when considering the influence of niche on the activity of CRISPR-Cas systems. Cell density is a fine example of such influence. Bacterial cells living in low density populations have relatively low risk of encountering invasive exogenous DNA, which is witnessed by lower expression of CRISPR-Cas systems. On the contrary, bacteria living in dense aggregations are susceptible to viral infections (Knowles et al., 2016) and horizontal gene transfer (Pinedo and Smets, 2005). To address this risk, CRISPR-Cas elements become upregulated in these conditions. Exception from this rule being bacteria that live in the biofilm. Although in high density, cells in biofilm are usually better protected from phages than in suspension (Hughes et al., 1998). Bacteria in biofilm tend to lower the activity of the system (Borges et al., 2020). This likely helps them to save resources, while still protected by various other protective mechanisms present in biofilm.

Another example of the niche having effect on regulation of CRISPR-Cas systems is related to the CAP protein. Studies have shown that intracellular and extracellular cAMP concentrations are modulated by adenylate cyclases, phosphodiesterases and cAMP efflux systems, some of which respond to external and/or internal signals. Changes in the intracellular concentration of cAMP are perceived by CAP proteins, which in turn respond with different change in activity of CRISPR-Cas. Type of that response is associated with different niches occupied by the given bacteria (Green et al., 2014). On the one hand, *E. coli* inhabiting human intestine increased CRISPR-Cas expression is observed at high glucose levels (Yang et al., 2014). This can be associated with increased metabolic activity of all gut bacteria after the meal, which may increase HTG (Rowland et al., 2018; Tan et al., 2022). On the other hand, saprotroph *P. atrosepticum* shows increased CRISPR expression at lower glucose concentrations (Patterson et al., 2015). This response may be a precaution taken to counter potential activation of prophages caused by starvation.

The other general pattern observed for regulation of CRISPR-Cas systems relates to maximizing the outcome of systems that are already active. An example of this being increased acquisition of new spacers induced by Csa3a protein (Liu et al., 2015) or the presence of AHL autoinducers (Patterson et al., 2016). With an updated CRISPR array being necessary for efficient interference, bacteria acquire new spacers whenever their general CRISPR-Cas machinery is upregulated. Observations made for bacteria like *P. aeruginosa* (Høyland-Kroghsbo et al., 2017) and *A. wodanis* (Maharajan et al., 2022) seem to confirm this.

The last but not the least, there is a pattern of downregulating CRISPR-Cas activity by NAPs such as H-NS, StpA and LRP (Herz and Strickland, 2001; Pul et al., 2010; Mitić et al., 2020). These proteins bind to DNA, which leads to a strong condensation, which in turn can inhibit gene transcription and reduce the metabolic activity of the cell. This may be because low activity bacteria are not the primary target of bacteriophages. However, it is also possible that downregulation of the CRISPR-Cas elements is nonspecific and happens as a part of a cell wide dormancy.

## 4. Current applications of CRISPR-Cas regulatory mechanisms

A milestone in the CRISPR-Cas research was the demonstration of the complementarity of the plasmid and phage sequences to the spacers of the CRISPR matrix. This was the first indication that this system could function as a prokaryotic defense mechanism (Bolotin et al., 2005; Pourcel et al., 2005; Mojica and Rodriguez-Valera, 2016). Since the discovery of the main function of the system, the amount of research in this area has increased dynamically, contributing to a better understanding of its mechanism of operation and the use of the CRISPR-Cas system as a next generation genome editing tool (Cong et al., 2013; Mali et al., 2013) and as a specific regulator of gene expression (Qi et al., 2013). The use of CRISPR-Cas to break the DNA continuity through double strand breaks can lead to two processes. The first is non-homologous end joining (NHEJ). This may result in frameshift mutations in the target gene, thereby interfering with its function or can lead to the deletion of all base pairs between the gaps in the DNA. The second process is homology directed repair (HDR), which only occurs in proliferating cells (Chu et al., 2015). Which enables the introduction of a specific desired change to a gene and may have important clinical implications (Rodríguez-Rodríguez et al., 2019). Another mechanism that exploits the CRISPR-Cas systems is the regulation of gene expression. Inactivation of both Cas9 nuclease domains produces a protein incapable of degrading DNA, but capable of repressing initiation or prolonging transcription under the control of RNA (Qi et al., 2013). The Cas complex can be further modified by fusion with specific activators or transcriptional repressors (Bikard et al., 2013; Cheng et al., 2013). The effects of regulation can be modulated by “multiple targeting” using multiple sgRNAs with sequences complementary to the entire promoter region of a given gene (Cheng et al., 2013). The uses of Cas9 proteins have been described for virtually all commonly studied eukaryotes, from yeast to human cells inclusive (Bikard et al., 2013; Cheng et al., 2013; Gilbert et al., 2013). In addition, DNA labeling and epigenome editing with CRISPR have been described (Hilton et al., 2015; Ma et al., 2015; Huang et al., 2017; Chen et al., 2018; Lau and Suh, 2018; Abid et al., 2021). In the context of genome editing, another protein, the Cas12 was also used. This protein was used, among others for genetic manipulation of plasmids, plasmid curing, supporting of point mutations, deletions, insertions, and replacements in cells. Studies have shown that Cas12 has a lower off-target tendency than Cas9 causes against bacterial (Yan et al., 2017), human (Kleinstiver et al., 2016) and plant cells (Ferenczi et al., 2017; Li et al., 2018).

Despite many advances the temporal and spatial regulation of CRISPR-Cas activity remains a challenge because excessive or prolonged nuclease activity may increase the likelihood of unplanned editing or may result in cell cytotoxicity. These side effects require a mechanism to disable for Cas9 once target edits are reached. When combined with modified Cas9 variants, the Acr proteins may act as an additional security feature to reduce potential adverse effects. The studies showed that the temporary delivery of AcrIIA4 to human cells in the form of an expression plasmid or single protein enables Cas9-mediated gene editing while reducing off-target editing (Shin et al., 2017). However, it was noted that this strategy has significant limitations. It is very sensitive in delivery efficiency. At 50% transfection rate, only half of the cells that received Cas9/gRNA in the first transfection will also receive Acr in the second transfection. In the other half, Cas9 would remain active, causing off-target effects. Moreover, the requirement of two separate, well planned delivery steps (one for Cas9-sgRNA and one for Acr) is difficult to implement in many application settings, e.g., in a therapeutic scenario. Therefore, another approach was tested, which used conjugation Cas9 to artificial inhibitory domains. The inhibition domains were artificially weakened Acr proteins (point mutations caused different inhibitory potency of Cas9) co-expressed with Cas9 or directly linked to Cas9 to fine-tune its activity to selected levels, thus achieving efficient kinetic isolation of the on- and off-target editing situations. This finding brings a highly comprehensive application approach to reducing the effects of CRISPR-Cas target exclusion through kinetic isolation (Aschenbrenner et al., 2020). It is also interesting to use Acr proteins to protect against genome editing from outside the target tissue by limiting Cas9 activity to selected tissue types (Hoffmann et al., 2019). A cell type specific Cas-on switch based on miRNA-regulated expression of Acr proteins was developed. The target sites for miR-122 or miR-1, which are abundant in liver and heart muscle cells, respectively, were inserted into the 3’UTR of Acr transgenes. Co-expression with Cas9 and sgRNA resulted in a knockdown of Acr and the release of Cas9 activity only in hepatocytes or cardiomyocytes, while Cas9 was effectively inhibited in other cells.

Nitrogen bases editors have been developed based on the CRISPR-Cas9 system. Since the background for most of verified human genetic diseases are point mutations, the primary edit can correct these disease-related mutations (Cooper and Krawczak, 1990; Chen and Knoepfler, 2016). Cas9 does not create double strand breaks and enables a precise, targeted point mutation in genomic DNA (Komor et al., 2016; Gaudelli et al., 2017; Zong et al., 2017; Rees et al., 2019). Cytosine base pair editors (CBE) can be used to convert CG base pair to TA base pair, and adenine base pair editors (ABE) convert AT base pair to GC base pair in a few nucleotides in a target (Komor et al., 2016; Gaudelli et al., 2017). However, recent studies have verified unwanted events such as small insertions or deletions (indels) in human (Hu et al., 2018; Koblan et al., 2018), animal (Ryu et al., 2018; Zuo et al., 2019), and plants cells (Kang et al., 2018; Jin et al., 2019). AcrIIA5 was shown to reduce unintentional effects during CRISPR-Cas9 modification of human cells (Liang et al., 2020).

Acr proteins can be modified for specific laboratory applications, e.g., a hybrid of AcrIIA4 with a light-induced LOV2 domain has been shown to control genome and epigenome editing mediated by *Streptococcus pyogenes* Cas9 and dSpyCas9 (nuclease deficient SpyCas9) – in optogenetics (Bubeck et al., 2018). Post-translational control of Acr proteins was achieved by fusing an inducible destabilization domain that degrades the protein in the absence of an external ligand - Shield1 ((1R)-3-(3,4-Dimethoxyphenyl)-1-[3-[2-(4-morpholinyl)ethoxy]phenyl]propyl(2S)-1-[(2S)-1-oxo-2-(3,4,5-trimethoxyphenyl)butyl]-2-piperidinecarboxylate; Nakamura et al., 2019). Modifications of the Acr protein also made it possible to use it as a relatively cheap and easy to implement Cas protein detection system in many laboratories. The advantages of this strategy include the highly specific interaction of Acr with the target Cas protein, as well as non-complicated production and modification of the Acr protein Acr proteins can provide alternatives to anti-Cas antibodies, facilitating the development of robust platforms for the detection, identification and quantification of the CRISPR-Cas system (Johnston et al., 2019). Another research group constructed a centrifugal microfluidic platform to measure both Cas9 protein level and nuclease activity using modified Acr proteins (Phaneuf et al., 2019).

The Acr proteins have also found use in the development of adenoviral vectors for the delivery of Cas9 to mammalian cells. To prevent self-cleavage during vector production, it was necessary to reduce the level of Cas9 mRNA as well as inhibit the activity of the Cas9 protein. Cas9 protein activity was inhibited by the expression of the Acr proteins (AcrIIA2 and AcrAII4) from both the producing cells and the helper virus (Palmer et al., 2019). Upon purification, these helper-dependent adenoviruses will perform self-cleavage in transduced target cells mediated by CRISPR-Cas9. This method significantly improved efficiency of vector production.

Another potential application of Acr proteins is the enhancement of phage therapies. These therapies are considered as an alternative to antibiotics in the treatment of bacterial infections (Nobrega et al., 2015). However, phage therapies may be inefficient against pathogenic hosts with active CRISPR-Cas systems such as *P. aeruginosa* (van Belkum et al., 2015) and *Neisseria meningitidis* (Zhang et al., 2019). Since Acr proteins have been found in these and other pathogens, the *acr* genes can be engineered into therapeutic bacteriophages that can inactivate CRISPR-Cas based anit-phage defences of these multi-drug resistant pathogenic bacteria. Although many Acrs were discovered to date they are likely to be the tip of the iceberg and many more of them will surely be described. Moreover, one can hypothesize that there are inhibitors of these fascinating new anti-phage systems have yet been discovered.

It is also worth mentioning about anti-CRISPR nucleic acids. Barkau and colleagues rationally designed small nucleic acid-based inhibitors, abbreviated as SNuBs, against CRISPR-Cas9 (Barkau et al., 2019). CRISPR SNuBs can inhibit enzyme activity (SpCas9) by 3 mechanisms: competing with the target DNA (anti-guide), or crRNA that pairs with tracrRNA (anti-tracr), or the PAM motif of targeted duplex DNA (anti-PAM). They concluded that an inhibitor must have an equal or greater affinity for SpCas9 than that SpCas9 has for crRNA, tracrRNA, or target DNA to ensure actual competition. The scientists also indicated potential developments of CRISPR SNuBs. They proposed that the binding of anti-tracr modules might be further improved by incorporating other RNA analogs to improve its interaction with Cas9. Taking into consideration that the anti-guide module showed little or no binding during tests, DNA or DNA analogues can be used to better mimic the target DNA. They discovered that relatively strong binding of Anti1_PAM resembles natural Acr protein (AcrIIA4), which interact with the PAM-interacting (PI) domain of Cas9 (Dong et al., 2017). However, anti-PAM modules might benefit from different sequence and structural designs, as well as chemical modifications that mimic DNA and improve nuclease resistance. According to their results, the combination of anti-tracr modules with anti-PAM modules, which form two different binding sites, may be important to achieve strong inhibition and high specificity. CRISPR SNuBs described in the study provide a platform for rational design of CRISPR-Cas enzyme inhibitors that should translate to other CRISPR effector enzymes and represents another possible control over CRISPR-based applications.

Another interesting example of the use of the regulation mechanism is the quorum detecting inhibitors that were proposed to be used to suppress the adaptive immune system of CRISPR-Cas in order to enhance medical applications, including phage therapies (Høyland-Kroghsbo et al., 2017). Making bacteria more prone to killing by phage therapy through inhibition of the CRISPR-Cas defense mechanism and quorum-sensing inhibitor should also reduce acquisition of resistance against the administered phage. Widespread resistance to antibiotics among bacteria makes the phage therapy a good alternative treatment. Moreover, a strategy known as quorum quenching (QQ), is of primary importance to simultaneously disorganize bacteria, reduce virulence, reduce biofilm formation and increase bacteriophage sensitivity (Mion et al., 2019). The effect of the QS interfering enzyme on the alteration of the regulation systems in clinical strains of *P. aeruginosa* as well as in the marine bacterium *Chromobacterium violaceum* CV12472 was observed. In most cases, expression of the CRISPR-Cas genes decreased suggesting that enzymatic disruption of QS is promising in modulating phage-bacterial interactions (Mion et al., 2019).

Other regulatory mechanisms have not yet been used to purposefully control CRISPR-Cas systems. Nevertheless, some proposals for utilization of mechanism affecting CRISPR-Cas systems have been made. One such application utilizes knowledge about the sensitivity of the CRISPR-Cas system to formation of nucleoid structure and DNA topology (Dorman and Ní Bhriain, 2020). The mechanism by which mobile genetic elements can suppress CRISPR-Cas transcription using H-NS homologues has also been proposed, and competition between H-NS or its homolog and their antagonist with the LeuO protein for access to the appropriate binding sites would provide the basis for a regulatory switch that could overcome transcription silencing *via* H-NS. As H-NS, IHF, LeuO, Lrp and variable DNA topology are involved in the regulation of virulence genes (Dillon et al., 2012; Ayala et al., 2017) there seems to be a relationship between CRISPR-Cas function, horizontal gene transfer and bacterial virulence.

## 5. Summary

**Figure 6:**
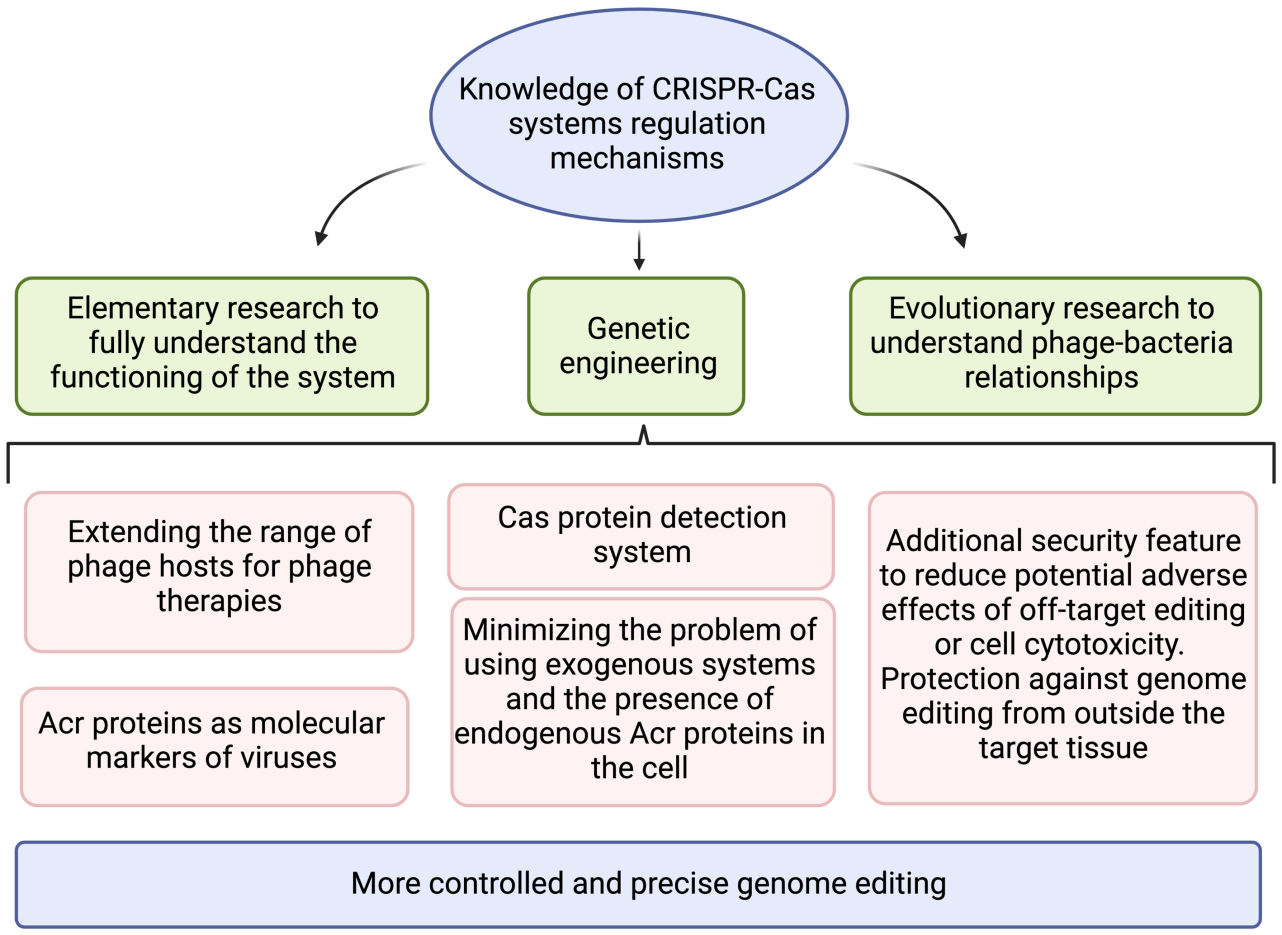
The summary of practical applications that benefit from increased knowledge on CRISPR-Cas regulation mechanisms. Created with BioRender.com.

The regulation of CRISPR-Cas systems can be based on both exogenous proteins of phage origin and on autoregulatory mechanisms. The constant arms race between phages and bacteria has resulted in development of a complex defense system, which is influenced by many factors. In addition, these systems are involved in the virulence of microorganisms by protecting them against the host’s immune response (Sampson et al., 2013; Li et al., 2016) or mediating the regulation of biofilm formation (Tang et al., 2019; Cui L. et al., 2020). Understanding the mechanisms of their regulation can significantly contribute to many fields such as the development of genetic engineering, it can increase the efficiency of the process of manipulating the genome of eukaryotic organisms, minimize the off-target events, making the better controlled CRISPR-Cas system successfully implemented in medical and biotechnological applications (Figure 6). Acr proteins can increase the accuracy and safety of CRISPR-based therapies, reduce the risk of uncontrolled effects, and reduce the likelihood of off-target cleavage (Shin et al., 2017). Moreover, the continual disclosure of Acr’s different mechanisms of action will help to better understand the relationship between bacteriophages and bacteria as they evolve (Liu et al., 2019). Without a doubt, there are plenty of discoveries to be made in this research area.

## Author contributions

MB conceived the manuscript. MB and MZ gathered data and revised the manuscript. MZ wrote the manuscript and prepared figures. All authors contributed to the article and approved the submitted version.

## Funding

This work was partially supported by the National Center of Science, Poland (grant no. 2020/04/X/NZ1/00309). Publication was financed by the University of Warsaw.

## Conflict of interest

The authors declare that the research was conducted in the absence of any commercial or financial relationships that could be construed as a potential conflict of interest.

## Publisher’s note

All claims expressed in this article are solely those of the authors and do not necessarily represent those of their affiliated organizations, or those of the publisher, the editors and the reviewers. Any product that may be evaluated in this article, or claim that may be made by its manufacturer, is not guaranteed or endorsed by the publisher.
